# Impact of obesity on breast cancer recurrence and minimal residual disease

**DOI:** 10.1186/s13058-018-1087-7

**Published:** 2019-03-13

**Authors:** Brett L. Ecker, Jun Y. Lee, Christopher J. Sterner, Aaron C. Solomon, Dhruv K. Pant, Fei Shen, Javier Peraza, Lauren Vaught, Samyukta Mahendra, George K. Belka, Tien-chi Pan, Kathryn H. Schmitz, Lewis A. Chodosh

**Affiliations:** 1 Department of Surgery, Perelman School of Medicine at the University of Pennsylvania, Philadelphia, PA USA; 2 Department of Cancer Biology, Perelman School of Medicine at the University of Pennsylvania, Philadelphia, PA USA; 3 2-PREVENT Translational Center of Excellence, Abramson Cancer Center, University of Pennsylvania, Philadelphia, PA USA; 4 The Abramson Family Cancer Research Institute, Perelman School of Medicine at the University of Pennsylvania, Philadelphia, PA 19104-6160 USA; 5 Penn State Cancer Institute, Penn State College of Medicine, Hershey, PA 17033 USA

**Keywords:** Obesity, High-fat diet, Breast cancer, Recurrence, Tumor dormancy

## Abstract

**Background:**

Obesity is associated with an increased risk of breast cancer recurrence and cancer death. Recurrent cancers arise from the pool of residual tumor cells, or minimal residual disease (MRD), that survives primary treatment and persists in the host. Whether the association of obesity with recurrence risk is causal is unknown, and the impact of obesity on MRD and breast cancer recurrence has not been reported in humans or in animal models.

**Methods:**

Doxycycline-inducible primary mammary tumors were generated in intact *MMTV-rtTA;TetO-HER2/neu* (*MTB/TAN*) mice or orthotopic recipients fed a high-fat diet (HFD; 60% kcal from fat) or a control low-fat diet (LFD; 10% kcal from fat). Following oncogene downregulation and tumor regression, mice were followed for clinical recurrence. Body weight was measured twice weekly and used to segregate HFD mice into obese (i.e., responders) and lean (i.e., nonresponders) study arms, and obesity was correlated with body fat percentage, glucose tolerance (measured using intraperitoneal glucose tolerance tests), serum biomarkers (measured by enzyme-linked immunosorbent assay), and tissue transcriptomics (assessed by RNA sequencing). MRD was quantified by droplet digital PCR.

**Results:**

HFD-Obese mice weighed significantly more than HFD-Lean and LFD control mice (*p* < 0.001) and had increased body fat percentage (*p* < 0.001). Obese mice exhibited fasting hyperglycemia, hyperinsulinemia, and impaired glucose tolerance, as well as decreased serum levels of adiponectin and increased levels of leptin, resistin, and insulin-like growth factor 1. Tumor recurrence was accelerated in HFD-Obese mice compared with HFD-Lean and LFD control mice (median relapse-free survival 53.0 days vs. 87.0 days vs. 80.0 days, log-rank *p* < 0.001; HFD-Obese compared with HFD-Lean HR 2.52, 95% CI 1.52–4.16; HFD-Obese compared with LFD HR 2.27, 95% CI 1.42–3.63). HFD-Obese mice harbored a significantly greater number of residual tumor cells than HFD-Lean and LFD mice (12,550 ± 991 vs. 7339 ± 2182 vs. 4793 ± 1618 cells, *p* < 0.001).

**Conclusion:**

These studies provide a genetically engineered mouse model for study of the association of diet-induced obesity with breast cancer recurrence. They demonstrate that this model recapitulates physiological changes characteristic of obese patients, establish that the association between obesity and recurrence risk is causal in nature, and suggest that obesity is associated with the increased survival and persistence of residual tumor cells.

**Electronic supplementary material:**

The online version of this article (10.1186/s13058-018-1087-7) contains supplementary material, which is available to authorized users.

## Background

Obesity is a growing health pandemic [1] whose global prevalence has more than doubled in the last quarter-century [2]. Excess body weight has been implicated in the etiology of a broad spectrum of human cancers [3, 4], including breast cancer, and recent projections suggest that obesity (defined by the World Health Organization [5] as a body mass index [BMI] ≥ 30.0 kg/m^2^) may exceed tobacco as the largest modifiable risk factor for cancer incidence in the United States [6]. Because breast cancer is already the most prevalent cancer in the United States and the leading cause of cancer mortality in women worldwide [7, 8], the impact of increases in the prevalence of obesity on breast cancer incidence and mortality is a major concern.

Of the 8 million female cancer survivors alive today in the United States, nearly half are breast cancer survivors [9]. Although long-term survival is generally favorable, up to 30% of breast cancer patients will eventually succumb to their disease, with the majority of deaths due to disease recurrence after a variable period of clinical remission following upfront multimodality therapy. Hence, most deaths resulting from breast cancer are due to an inability to effectively prevent tumor recurrence [10]. Similarly to epidemiological data linking obesity with increased primary breast cancer risk, obesity is also associated with a greater risk of breast cancer recurrence [11–16] (HRs ranging from 1.17 to 1.46 [13–15]), as well as a 30–40% increased risk of breast cancer-related death [11, 15, 17, 18]. Because the prevalence of obesity among U.S. women is > 40% [19] and may be even higher among breast cancer survivors [20], understanding the biological underpinnings of the association between obesity and recurrence risk is a critical unmet need.

The association between obesity and increased risk of breast cancer recurrence and death reflects a variety of biological and nonbiological factors, including delayed detection [21], more advanced presentation at diagnosis [11, 14, 22], and the use of suboptimal doses of chemotherapeutic agents relative to body size [17, 23], as well as increased risks of second primary cancers and non-cancer-related causes of death [24]. Notwithstanding these factors, the negative impact of obesity on recurrence and long-term survival persists independently of biases related to detection, primary disease burden, and treatment [14, 18, 25], and it has been observed irrespective of menopausal status [11, 18, 25–27] and across a spectrum of breast cancer subtypes [17, 18, 25, 28].

Obesity is associated with dysregulation of multiple biologic pathways involving adipokines, insulin and insulin-like growth factor (IGF) signaling, endogenous sex hormone levels, and chronic inflammation [29]. Despite epidemiologic evidence suggesting a role for each of these pathways in obesity-accelerated recurrence, mechanistic support for the hypothesized causal relationship between obesity and breast cancer has been derived almost exclusively from animal models for primary, rather than recurrent, tumorigenesis. In this regard, increasing evidence suggests that the pathways that drive breast cancer recurrence are likely to be distinct from those associated with primary tumorigenesis [30–34]. Consequently, the mechanisms by which obesity affects breast cancer recurrence may differ from those involved in primary breast cancer development.

Rodent models of diet-induced obesity have been essential for demonstrating a causal relationship between obesity and accelerated primary tumorigenesis [35–48]. However, because the vast majority of breast cancer deaths are due to recurrence, understanding the mechanistic basis for the association of obesity with tumor recurrence is essential. Unfortunately, progress toward this goal has been limited by inherent challenges in human trial design and the absence of an appropriate preclinical animal model.

To address this important gap, we have employed a previously validated transgenic mouse model for breast cancer recurrence to test the causal relationship between obesity and mammary tumor recurrence [30–32, 49]. *MMTV-rtTA;TetO-HER2/neu* (*MTB/TAN*) mice conditionally express the *HER2/neu* oncogene and develop invasive mammary adenocarcinomas in a tissue-specific manner in response to chronic induction with doxycycline [49, 50]. Following oncogene downregulation and pathway inhibition by doxycycline withdrawal, mammary tumors regress to a nonpalpable state in a manner analogous to the treatment of cancers with targeted therapies such as trastuzumab [51]. However, a small population of residual tumor cells persist following tumor regression and reside in a dormant state [30–32, 52]. Moreover, as occurs in patients with breast cancer, spontaneous local and distant recurrences arise from this reservoir of residual tumor cells following a variable period of latency [30–32, 49, 52, 53].

The clinical relevance of the *MTB/TAN* genetically engineered mouse model is supported by several key findings. In particular, functional interrogation of this model has identified several pathways that contribute to tumor recurrence in mice, including NOTCH [31], SPSB1 [30], SNAIL [54], CERK [52], and PAR-4 [32], each of which is strongly associated with risk of distant relapse in patients with breast cancer and in the direction predicted by studies in mice, as well as in a manner that is neither specific for local relapse nor restricted to a particular subtype of breast cancer. Furthermore, survival of minimal residual disease (MRD) in the mouse mammary gland following chemotherapy or targeted therapy parallels that of patients who receive neoadjuvant therapy but do not achieve pathological complete response. Indeed, in both mice and humans, survival of local residual tumor cells in the mammary gland following therapy is prognostic for relapse at distant sites [55, 56]. Also of note, recurrent tumors arising in *MTB/TAN* mice often lack human epidermal growth factor receptor 2 (HER2) overexpression, such that recurrence is driven by the activation of alternate growth and survival pathways [30–32, 52–54, 57, 58]. This is paralleled by clinical observations that HER2+ primary breast cancers in patients frequently give rise to HER2− residual disease [59–61] and HER2− recurrent tumors [61]. This strongly suggests that residual tumor cells can survive and recur via HER2-independent pathways in both mice and humans. Finally, residual disease and recurrent tumors in *MTB/TAN* mice often exhibit a triple-negative (estrogen receptor [ER]-negative, progesterone receptor [PR]-negative, HER2-negative) phenotype, which in patients is associated with an increased risk of recurrence [62, 63]. In aggregate, these findings indicate that the biology of the *MTB/TAN* model is neither specific for nor restricted to a particular subtype of human breast cancer, and is neither specific for nor restricted to local as opposed to distant sites of recurrence. This, in turn, supports the clinical relevance of this model and suggests that it is informative for the biology of residual tumor cells that survive selection pressures imposed by targeted therapy or the microenvironment at local or distant sites.

In the present study, *MTB/TAN* mice fed a high-fat diet developed phenotypic and physiologic features characteristic of human obesity, including excess body fat, hyperinsulinemia, impaired glucose tolerance, and dysregulated circulating adipokines. Consistent with this, obese mice experienced an accelerated rate, as well as an increased frequency, of mammary tumor recurrences compared with lean mice, and this was associated with the survival and persistence of an increased number of residual tumor cells in obese mice. These studies provide a preclinical model in which to study the association of obesity with breast cancer recurrence, demonstrate that this association is causal in nature, and suggest that it is associated with obesity-induced alterations in the survival and persistence of residual tumor cells.

## Methods

### Animals and orthotopic recurrence assays

All mouse experiments were performed in accordance with guidelines of the Institutional Animal Care and Use Committee (protocol number 803351) at the University of Pennsylvania (Philadelphia, PA, USA). All transgenic lines were created and maintained on an inbred *FVB/N* background. Bitransgenic *MMTV-rtTA;TetO-HER2/neu* (*MTB/TAN*) mice were generated by cross-breeding, and tumors were generated by doxycycline induction of *HER2* transgene expression, as described previously [31, 49]. Orthotopic recurrence assays in monotransgenic *TetO-HER2/neu* (*TAN*) mice were performed as described in *nu/nu* mice and were carried out by injecting 1 × 10^6^ *MTB/TAN* primary tumor cells into the number 4 mammary fat pad of recipient *TAN* mice maintained on doxycycline. In contrast to the *MTB/TAN* model, *TAN* mice do not induce the *HER2* transgene or form tumors when exposed to doxycycline; however, the similar genetic background permits engraftment of doxycycline-sensitive *MTB/TAN* tumor cells into the *TAN* host.

For both *MTB/TAN* and *TAN* mice, primary tumors formed in the presence of HER2 signaling, after which oncogene downregulation induced by doxycycline withdrawal was used to simulate targeted therapy. All tumors regressed to a nonpalpable state, and mice were followed twice weekly until clinical recurrence or 250 days in the absence of recurrence [31, 32]. Study diets included a high-fat diet (HFD; OpenSource D12492 [Research Diets Inc., New Brunswick, NJ, USA]: 60%, 20%, and 20% of calories from fat, carbohydrate, and protein, respectively) or a low-fat diet (LFD; OpenSource D12450B [Research Diets Inc.]: 10%, 70%, and 20% calories from fat, carbohydrate, and protein, respectively). The fat content of the HFD was derived from lard and thus was high in saturated fats. Mice were fed ad libitum.

### Biometric parameters

The body weight of each mouse was measured twice weekly throughout the study. Body composition (total, fat and lean mass) was measured using the EchoMRI™ 3-in-1 Body Composition Analyzer (EchoMRI, Houston, TX, USA).

### Glucose tolerance testing

Intraperitoneal glucose tolerance tests (IP-GTTs) were performed following complete primary tumor regression (i.e., 4 weeks following doxycycline de-induction) and at the time of clinical recurrence. A glucose bolus (10 μl/g body weight of 20% glucose solution) was administered following a 16-h overnight fast, and blood samples were measured by glucometer from the tail vein at 0, 30, 60, 90, and 120 minutes.

### Quantification of serum biomarkers

Serum was collected by cardiac puncture 10 weeks following primary tumor regression, and selected biomarkers were evaluated by enzyme-linked immunosorbent assay (ELISA) according to manufacturers’ protocols. All samples were run in duplicate. The adipokine multiplex panel (insulin, leptin, resistin, tissue plasminogen activator 1) was assessed using the MADKMAG-71K assay kit (EMD Millipore, Billerica, MA, USA). Adiponectin levels were assessed using the EZMADP-60K assay kit (EMD Millipore). IGF-1 and insulin-like growth factor-binding proteins 1–7 (IGFBP1–7) were assessed using the 22-IG1MS-E01 assay kit (ALPCO, Salem, NH, USA) and the MIGFBPMAG-43K multiplex kit (EMD Millipore), respectively. Estradiol, testosterone, and sex hormone-binding globulin were measured using the ES180S-100 (Calbiotech, El Cajon, CA, USA), 55-TESMS-E01 (ALPCO), and CSB-E08233m (Biotrend, Destin, FL, USA) assays, respectively. Hepatocyte growth factor (HGF) was measured using the MHG00 assay kit (R&D Systems, Minneapolis, MN, USA). The inflammatory markers C-reactive protein (CRP), monocyte chemoattractant protein (MCP)-1, and corticosterone were assessed using the RH971CRP01MR (BioVendor, Brno, Czech Republic), MJE00 (R&D Systems), and 55-CORMS-E01 (ALPCO) assay kits, respectively. Multiplex ELISA plates were read on a Luminex 100 plate reader (Luminex Corp., Austin, TX, USA) and analyzed using Exponent software (Texture Technologies, Hamilton, MA, USA). Single ELISAs were read on the Molecular Devices M2 plate reader and analyzed using SoftMax Pro software (Molecular Devices, Sunnyvale, CA, USA).

Because of the low concentration of tumor necrosis factor (TNF)-α and interleukin (IL-6) in serum, these two cytokines were measured using single-molecule arrays (Simoa), an ultrasensitive ELISA technique (Quanterix, Lexington, MA, USA) [64]. Serum was collected from mice with MRD at 4 weeks after de-induction, data were acquired using Simoa HD-1 Analyzer software version 1.5 (Quanterix) and analyzed using Excel (Microsoft, Redmond, WA, USA) and Prism 7 (GraphPad Software, La Jolla, CA, USA) software.

### RNA isolation, qRT-PCR, and RNA sequencing

RNA was isolated from tissues using TRIzol reagent (Life Technologies/Thermo Fisher Scientific, Carlsbad, CA, USA) followed by the RNeasy Mini Kit (Qiagen, Valencia, CA, USA). Reverse transcription was performed using the High-Capacity cDNA Reverse Transcription Kit (Applied Biosystems/Thermo Fisher Scientific, Foster City, CA, USA) according to the manufacturer’s instructions. qPCR was performed using the ViiA 7 Real-Time PCR System (Life Technologies) using 6-carboxyfluorescein (FAM)-labeled TaqMan probes for ErbB2 (Rn00566561_m1) and Tbp (Mm00446973_m1) (Applied Biosystems). Expression levels were normalized to Tbp.

For RNA sequencing (RNA-Seq), samples were processed using the High-Throughput Sequencing Core at the Children’s Hospital of Philadelphia in collaboration with Beijing Genomics Institute. RNA-Seq was performed on the HiSeq 4000 platform (Illumina, San Diego, CA, USA) with 30 million 100-bp paired-end reads per sample. The quality of raw data was evaluated by FastQC [65] and reads from samples passing quality control parameters were aligned to the mm10 mouse reference genome using the STAR aligner [66]. Aligned reads were counted at the gene level using featureCounts [67]. Normalization of read counts and differential expression analysis between sample groups were performed using DESeq2 [68].

A mouse obesity signature was generated by comparing RNA-Seq data from parametrial fat tissues of HFD-Obese and LFD mice (*n* = 6/arm). The signature was defined as the 679 genes differentially expressed between the two arms using DESeq2 at a fold change cutoff > 1.5 and a false discovery rate cutoff < 0.1. Signature scores were defined as weighted mean expression of the 679 signature genes in a BMI-stratified human dataset (Gene Expression Omnibus [GEO] accession number GSE27949), where the weights were 1 for genes higher in expression in HFD-Obese mice and − 1 for genes higher in expression in LFD mice. Expression levels in GEO accession number GSE27949 were first log_2_-transformed and then standardized by gene to a mean of 0 and an SD of 1 before signature score calculation. Mapping between mouse and human genes was done using HomoloGene build 68.

### Droplet digital PCR

DNA was extracted from the fourth mammary gland of killed *TAN* mice following primary tumor regression. Total DNA was purified according to the manufacturer’s instructions (DNeasy Blood & Tissue Kit, catalogue number 69506; Qiagen). Briefly, tissue specimens were homogenized in 180 μl of Buffer ATL (Qiagen) and 20 μl of proteinase K and incubated at 56 °C until completely lysed. Subsequently, 200 μl of Buffer AL and 200 μl of ethanol (100%) were added to the tissue lysate, and the mixture was centrifuged through DNeasy Mini spin columns (6000 × *g* for 1 minute). The flow-through was discarded, and centrifugation was repeated twice using 500 μl of Buffer AW1 (Qiagen) followed by 500 μl of Buffer AW2 (Qiagen). The DNeasy Mini spin column was moved to a clean 2-ml microcentrifuge tube, and 100 μl of Buffer AE (Qiagen) was added directly onto the DNeasy membrane and centrifuged (6000 × *g* for 1 minute) for elution.

Droplet digital PCR (ddPCR) was performed using the RainDrop Plus™ System (RainDance Technologies, Billerica, MA, USA). The reaction was carried out in a final volume of 30 μl, which included 15 μl of TaqMan™ Genotyping Master Mix (catalogue number 4371355; Applied Biosystems), 1.5 μl of *rtTA* primers/probe mix 20× (TaqMan probe labeled with FAM; Applied Biosystems), 1.2 μl of droplet stabilizer 20× (catalogue number 30-07026; RainDance Technologies), and 10 μl of sample nucleic acid solution (sample prediluted to 100 ng/μl) and H_2_O to reach the final volume. Reaction mixtures were placed into the sample wells of the RainDrop Source chip (RainDance Technologies), and droplets were formed in the RainDrop Source droplet generator (RainDance Technologies). After processing, the droplets were transferred to a thermal cycler for PCR amplification with the following thermal profile: hold at 95 °C for 10 minutes, 45 cycles of 95 °C for 15 seconds and 60 °C for 1 minute (ramp 0.5 °C/second), 1 cycle at 98 °C for 10 minutes, and hold indefinitely at 12 °C. After amplification, the Sense chip was loaded on the RainDrop Sense droplet reader (RainDance Technologies) to read droplets in all sample wells. RDA II software (RainDance Technologies) was used to provide absolute quantification of target DNA.

### Statistical analyses

Unpaired Student’s *t* tests were used to analyze normally distributed data. Mann-Whitney *U* tests were used when data were not normally distributed. Two-way analysis of variance was used to compare paired data. Log-rank tests were used to analyze survival curves. *P* values < 0.05 were considered statistically significant. HRs with 95% CIs were calculated for all survival curves.

## Results

### *FVB/N* mice are susceptible to diet-induced obesity

To test the relationship between diet-induced obesity and mammary tumor recurrence in mice, we employed the *MTB/TAN* model of mammary tumor recurrence previously validated in our laboratory [30–32, 49]. Notably, this model was created in and maintained on an *FVB/N* genetic background, which has been reported to be resistant to diet-induced obesity. To determine whether an obese phenotype could be generated in *MTB/TAN* mice on an *FVB/N* background, 3-week-old female mice were randomized to a control LFD, consisting of 10% calories from fat, or to a matched HFD consisting of 60% calories from fat, which is comparable to the > 50% fat content of an In-N-Out® double cheeseburger (In-N-Out, Irvine, CA, USA), a Pizza Hut® Meat Lover’s pizza (Pizza Hut, Plano, TX, USA), or a loaded baked potato.

**Fig. 1:**
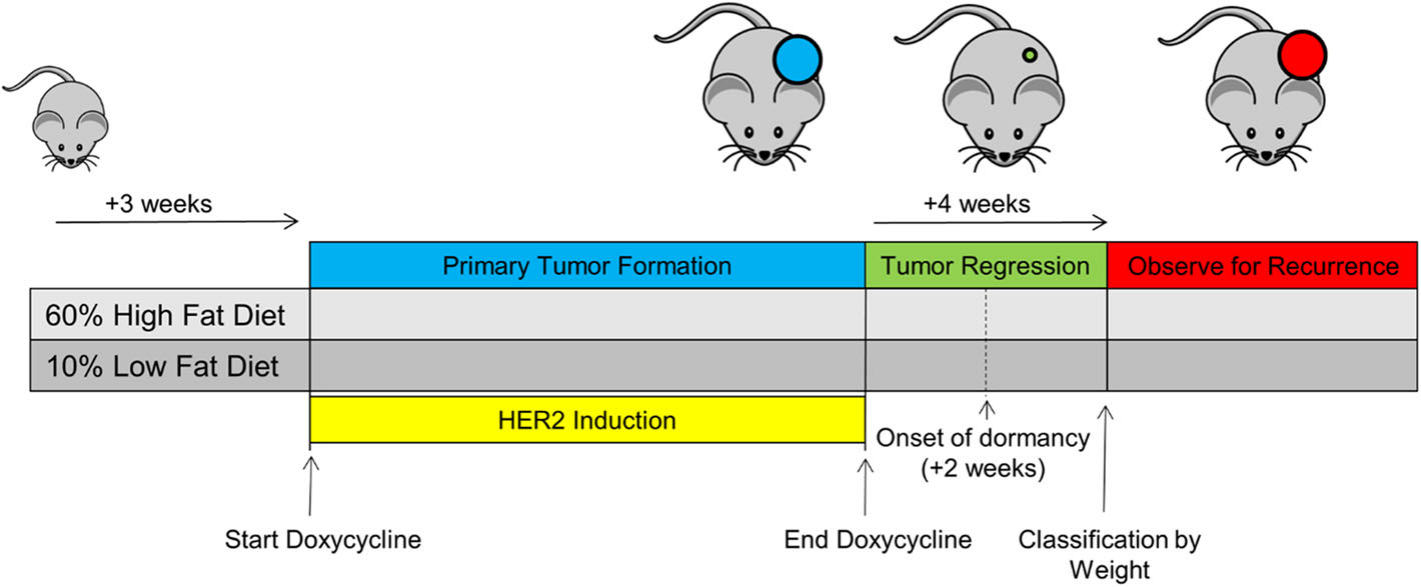
Study design. Three-week-old female *MTB/TAN* mice were weaned onto matched study diets consisting of 60% calories from fat (high-fat diet) or 10% calories from fat (low-fat diet). Doxycycline (0.03 mg/ml) was provided in drinking water beginning at age 6 weeks to induce human epidermal growth factor receptor 2 (HER2)-driven mammary tumors. Once primary tumors reached target volume (500 cm^3^), the *HER2* oncogene was downregulated by doxycycline withdrawal to induce tumor regression. Mice were then observed twice weekly for recurrence

HFD and LFD were provided in the context of a mammary tumor recurrence assay (Fig. 1). In brief, doxycycline was provided to HFD- and LFD-fed mice beginning at 6 weeks of age to drive oncogene-dependent primary breast tumorigenesis, following which tumor regression was induced by doxycycline withdrawal. Study “enrollment” was defined as the time point at which mice were classified as obese or lean, which occurred 4 weeks following oncogene de-induction and corresponded to a point at which primary tumors had completely regressed, leaving behind a small population of dormant residual tumor cells. Obese and nonobese mice were then monitored for tumor recurrence to determine the impact of obesity.

**Fig. 2:**
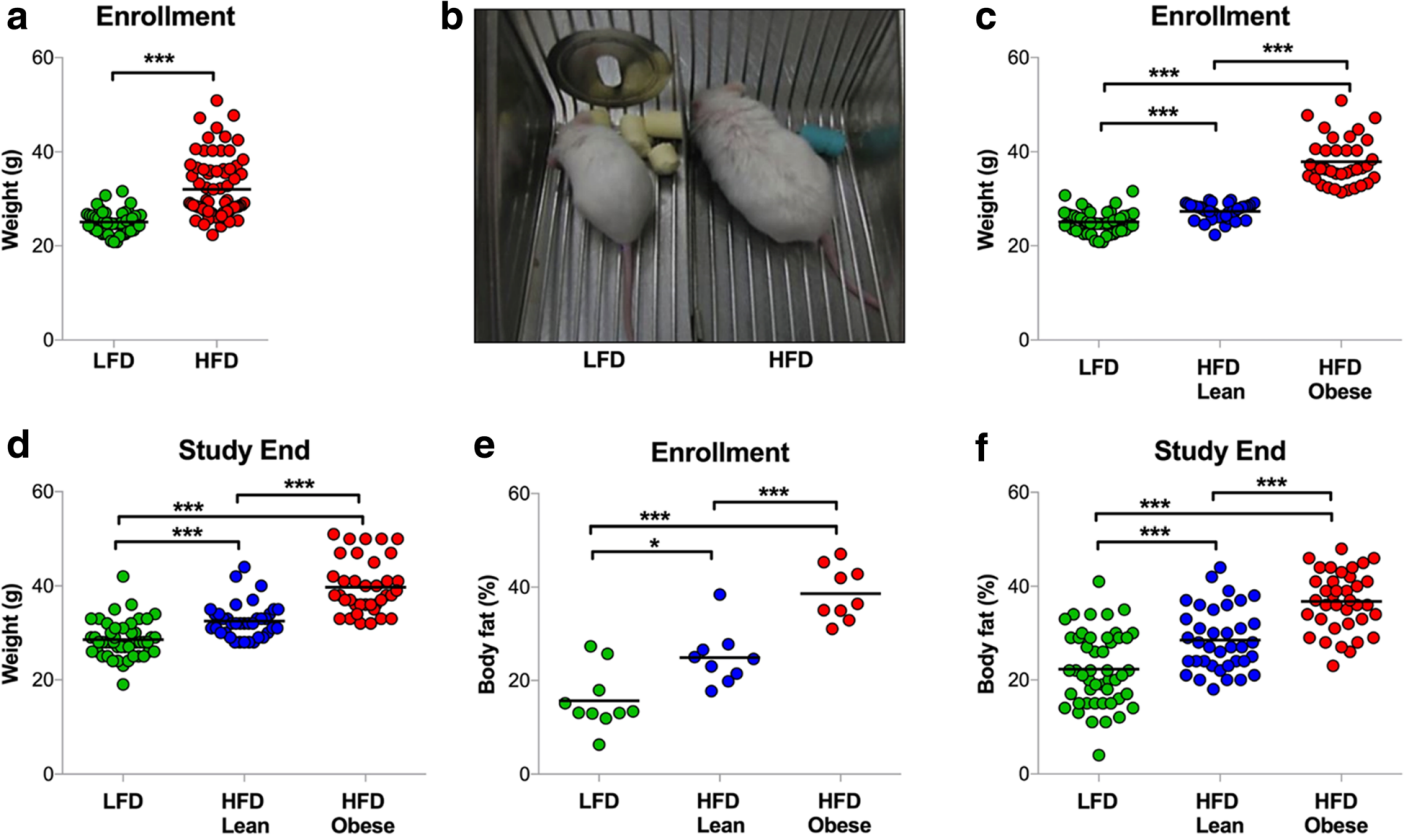
**a** Body weights were measured in *MTB/TAN* mice fed a high-fat diet (HFD) or low-fat diet (LFD) 4 weeks following tumor regression induced by doxycycline withdrawal. HFD mice weighed significantly more than LFD mice (32.9 ± 6.8 g vs. 25.2 ± 2.6 g, *p* < 0.001). **b** Representative photograph of mice fed a LFD or HFD. **c** HFD-Obese mice weighed more than HFD-Lean and LFD mice at enrollment (38.2 ± 5.1 g vs. 27.2 ± 1.8 g vs. 25.2 ± 2.6 g, respectively; *p* < 0.001; *n* = 50/arm). **d** HFD-Obese mice remained heavier than HFD-lean and LFD mice at study end (39.7 ± 5.4 g vs. 32.2 ± 3.9 g vs. 28.5 ± 3.9 g, respectively; *p* < 0.001; *n* = 50/arm). **e** Four weeks following doxycycline withdrawal, body fat composition determined by nuclear magnetic resonance was significantly increased in HFD-Obese mice compared with HFD-Lean and LFD mice, with greater total fat mass (14.3 ± 3.7 g vs. 6.9 ± 2.3 g vs. 4.2 ± 2.1 g, respectively; *p* < 0.001; *n* = 10/arm) and body fat percentage (38.6 ± 5.8% vs. 24.9 ± 6.0% vs. 15.7 ± 6.4%, respectively; *p* < 0.001). **f** HFD-Obese mice had greater body fat percentage than LFD and HFD-Lean mice at study end (36.8 ± 6.4% vs. 28.5 ± 6.7% vs. 22.4 ± 8.0%, *p* < 0.001; *n* = 50/arm). Figures are dot plots showing mean data. *p < 0.05 and ***p < 0.001

As a group, mice fed an obesogenic diet were significantly heavier at the time of enrollment into the recurrence assay (total body weight 32.9 ± 6.8 g vs. 25.2 ± 2.6 g, *p* < 0.001) (Fig. 2a, b). Notably, genetically identical mice exhibited varying responses to challenge with a HFD, with some becoming obese and others maintaining weights comparable to LFD controls (Fig. 2a). To define obesity in a manner analogous to humans, mice that were 20% heavier at the time of enrollment relative to age-matched LFD mice were defined as “HFD-Obese”, since this roughly corresponds to the average weight difference between a BMI of 24.9 kg/m^2^ (normal) and a BMI of 30 kg/m^2^ (obese) for U.S. females of average height [69]. Approximately half of the cohort of HFD mice exhibited body weights that were 20% heavier than age-matched LFD mice. In contrast, HFD mice whose body weights were < 20% greater than age-matched LFD mice were classified as “HFD-Lean.” The use of diet responders (HFD-Obese) and nonresponders (HFD-Lean) provided the opportunity to study the influence of obesity apart from the potentially confounding effects of a HFD per se.

HFD-Obese mice weighed significantly more than LFD and HFD-Lean mice at enrollment (Fig. 2c), and at study end (Fig. 2d). Increased body weight corresponded to greater fat mass, with a 22% absolute increase in body fat percentage observed for HFD-Obese compared with LFD mice at time of enrollment (*p* < 0.001) (Fig. 2e), which persisted at study end (Fig. 2f). This approximates the 15% absolute difference in body fat percentage between lean (BMI < 25 kg/m^2^) and obese (≥ 30 kg/m^2^) women observed in the National Health and Nutrition Examination Survey III study [45]. These observations indicate that some *FVB/N* mice fed a HFD are susceptible to diet-induced obesity.

### Obesity alters serum adipokines

**Fig. 3:**
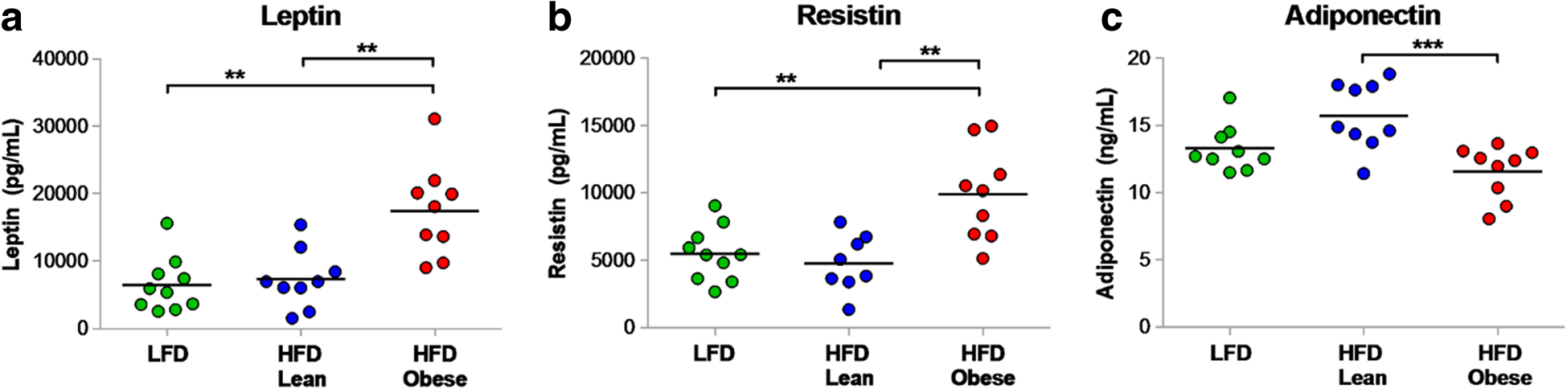
**a** Serum adipokines were measured by enzyme-linked immunosorbent assay in a subset of mice (*n* = 10/arm) at time of enrollment. High-fat diet (HFD)-Obese mice had higher circulating levels of leptin relative to HFD-Lean and low-fat diet (LFD) mice (17,512 ± 6876 pg/ml vs. 7310 ± 4341 pg/ml vs. 6461 ± 4036 pg/ml, respectively; *p* < 0.001). **b** Serum resistin levels were elevated in HFD-Obese mice relative to HFD-Lean and LFD mice (9884 ± 3443 pg/ml vs. 6158 ± 4653 pg/ml vs. 5486 ± 2008 pg/ml, respectively; *p* < 0.001). **c** Serum adiponectin levels were decreased in HFD-Obese mice relative to HFD-Lean and LFD mice (11.6 ± 2.0 ng/ml vs. 15.7 ± 2.5 ng/ml vs. 13.0 ± 1.8 ng/ml, respectively; *p* < 0.001). Figures are dot plots showing mean data. **p < 0.01 and ***p < 0.001

Hallmarks of human obesity include increased weight and fat mass [70], as well as dysregulation of adipokine signaling. Circulating concentrations of adipokines, such as leptin, resistin, and adiponectin, are closely tied to the proportion of body fat and have been implicated in the regulation of cellular growth, proliferation, and apoptosis [71–73]. To determine the effect of diet-induced obesity on serum adipokine expression, a subset of mice (*n* = 10/arm) was killed following enrollment into the recurrence assay, and serum adipokine levels were measured by ELISA. Analogous to observations in humans, HFD-obese mice exhibited higher levels of leptin and resistin (Fig. 3a, b), which are associated with excess fat in humans and implicated in poorer breast cancer prognosis [74–80], as well as lower levels of adiponectin (Fig. 3c), an adipose tissue secretory protein inversely correlated with body fat and inversely associated with breast cancer risk [78, 81]. These findings indicate that obesity in *FVB/N* mice is accompanied by changes in adipokine levels characteristic of obesity in humans.

### Glucose tolerance and obesity-associated hyperinsulinemia

**Fig. 4:**
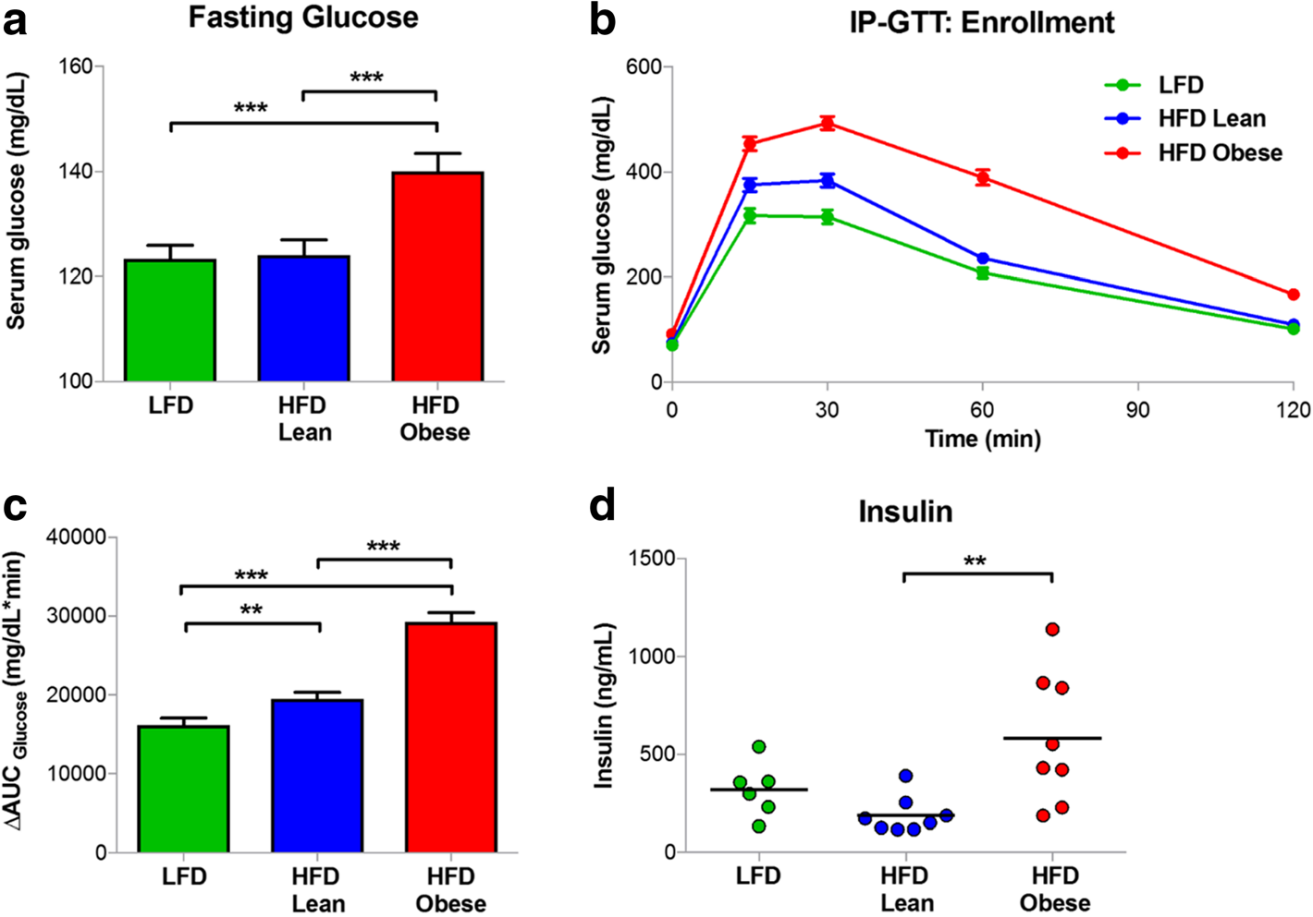
**a** Fasting serum glucose levels were measured after an overnight fast at time of enrollment. Obesity was associated with elevated fasting serum glucose levels relative to high-fat diet (HFD)-Lean and low-fat diet (LFD) mice (140.0 ± 24.7 mg/dl vs. 124.1 ± 19.8 mg/dl vs. 123.3 ± 18.9 mg/dl, respectively; *p* < 0.001; *n* = 50/arm). **b** Glucose values during 2-h intraperitoneal glucose tolerance test (IP-GTT). **c** Quantification of 2-h IP-GTT assay using the AUC of serum glucose levels as a function of time. HFD-Obese mice had significantly higher serum glucose levels following a glucose load, relative to HFD-Lean and LFD mice (30,500.4 ± 7437.7 mg/dl/minute vs. 19,798.4 ± 5419.4 mg/dl/minute vs. 16,557.1 ± 6517.3 mg/dl/minute, respectively; *p* < 0.001; *n* = 50/arm). **d** Fasting serum insulin was measured by enzyme-linked immunosorbent assay in a subset of mice (*n* = 10/arm) at time of enrollment. HFD-Obese mice exhibited hyperinsulinemia relative to HFD-Lean and LFD mice (519.9 ± 366.1 ng/ml vs. 168.3 ± 106.8 ng/ml vs. 405.6 ± 257.3 ng/ml, respectively; *p* = 0.028). Dot plots show mean data. Error bars represent the SEM. **p < 0.01 and ***p < 0.001

Obesity in humans is frequently associated with impaired insulin sensitivity [82]. To determine if increased body fat in HFD-Obese mice was associated with altered glucose tolerance, serum glucose levels were measured following an overnight fast at time of enrollment into the recurrence assay. HFD-Obese mice exhibited elevated fasting glucose levels compared with LFD or HFD-Lean mice (Fig. 4a). To assess glucose tolerance, fasted mice were administered an intraperitoneal glucose load (IP-GTT). HFD-Obese mice exhibited impaired responses to a glucose load, as evidenced by an increased AUC for the 2-h assay (Fig. 4b, c). Elevated fasting glucose and impaired glucose tolerance were accompanied by elevations in circulating insulin levels in fasting HFD-Obese mice (Fig. 4d).

Normalization of glucose tolerance has been reported in certain mouse models of diet-induced obesity following prolonged exposure to an obesogenic diet [83]. Therefore, to evaluate whether obese *MTB/TAN* mice displayed persistent impairment in glucose tolerance at the time of tumor recurrence, IP-GTT was repeated at study end. HFD-Obese mice displayed significantly elevated fasting glucose levels compared with LFD mice, with similar trends compared with the HFD-lean arm (Additional file 1: Figure S1a). Similarly, HFD-Obese mice exhibited decreased glucose tolerance compared with LFD and HFD-Lean mice (Additional file 1: Figure S1b, c). These results indicate that obesity in *FVB/N* mice is accompanied by fasting hyperglycemia, hyperinsulinemia, and decreased glucose tolerance, suggesting insulin resistance, as is characteristically observed in obese individuals.

### Serum biomarkers of obesity

Dysregulation of a number of pathways has been hypothesized to mechanistically link obesity with increased risk of breast cancer recurrence in humans, including changes in circulating adipokines, insulin/IGF-1, sex hormones, and chronic inflammation [29, 84–86]. In addition to the significant differences observed in serum adipokines, fasting glucose levels, insulin levels, and glucose tolerance, obesity was also associated with elevated serum levels of IGF-1, IGF-1/IGFBP2, and IGF-1/IGFBP3 in HFD-Obese mice (Table 1). IGF-1 levels increased progressively in mice exposed to a HFD, with the highest levels observed in HFD-Obese mice, though levels were significantly elevated in both HFD-Obese and HFD-Lean mice compared with LFD mice. Obesity in this model was not associated with significant increases in circulating levels of 17β-estradiol or testosterone, or in circulating levels of inflammatory markers, including CRP, tissue plasminogen activator inhibitor 1, TNF-α, MCP, IL-6, or corticosterone (Table 1). In summary, the serological and hormonal profile of obese *MTB/TAN* mice suggests the adipokine and insulin/IGF-1 signaling pathways as potential mediators of a relationship between obesity and breast cancer recurrence in this model.

**Table 1:** Serum biomarkers of diet-induced obesity (*n* = 10/arm)

	LFD	*p* Value^a^ vs. HFD-Obese	HFD-Lean	*p* Value^b^ vs. HFD-Obese	HFD-Obese	*p* Value^c^ across arms
Sex hormones						
Estradiol, pg/ml	5.46 [4.97–6.49]	0.190	4.82 [4.22–7.55]	0.739	4.81 [4.4–5.83]	0.474
Testosterone, ng/ml	0.492 [0.161–0.754]	0.463	0.466 [0.124–7.17]	0.876	0.227 [0.197–0.44]	0.769
SHBG, μg/ml	1.3 [1.08–303]	0.631	24.7 [1.44–332]	0.529	9.65 [1.15–316]	0.416
Adipokines						
Leptin, pg/ml	5630 [3190–8070]	**< 0.001**	6940 [4250–12,100]	**< 0.001**	18,100 [11,800–20,900]	**< 0.001**
Adiponectin, ng/ml	12.6 [11.6–14.1]	0.190	14.7 [13.4–17.9]	**0.002**	12.2 [9.96–13]	**0.006**
Resistin, pg/ml	5410 [3640–6690]	**0.004**	5640 [3600–7780]	**0.023**	9250 [6950–12,400]	**0.012**
Inflammatory markers						
TNF-α, pg/ml	9.86 [5.67–12.5]	0.268	11.6 [10.1–14.5]	0.918	12.5 [8.33–15.2]	0.287
IL-6, pg/ml	4.58 [2.13–5.94]	0.536	0.548 [0.387–1.13]	0.083	1.88 [0.316–11.6]	**0.035**
MCP-1, pg/ml	39.3 [36.9–41.9]	0.094	38.7 [31.1–53.2]	0.202	64.4 [40.2–93.2]	0.175
CRP, ng/ml	18.2 [15.3–19.4]	0.286	16.9 [15.2–18.1]	0.421	15.7 [15–18]	0.334
tPAI-1, pg/ml	12,200 [8650–16,200]	0.739	11,900 [9480–14,900]	0.481	13,900 [8580–17,500]	0.774
Corticosterone, ng/ml	110 [72.5–164]	0.315	200 [85.2–304]	0.796	199 [75.3–223]	0.379
HGF, pg/ml	9910 [7860–10,800]	0.280	9370 [7600–11,900]	0.247	8520 [6210–10,300]	0.384
Insulin/IGF-1 signaling						
Insulin, pg/ml	359 [216–625]	0.481	161 [117–245]	**0.011**	426 [228–840]	**0.020**
IGF-1, ng/ml	462 [417–542]	**< 0.001**	542 [518–609]	0.280	581 [559–617]	**0.002**
IGFBP1, ng/ml	7.12 [4.35–11.5]	0.064	5.16 [4.1–9.01]	0.436	4.37 [3.32–7.02]	0.170
IGFBP2, ng/ml	145 [107–184]	0.912	224 [155–292]	**0.019**	148 [105–176]	0.057
IGFBP3, ng/ml	204 [191–213]	0.579	197 [176–223]	0.912	193 [176–228]	0.836
IGFBP5, ng/ml	12.7 [0.811–18.8]	0.345	6.97 [0.861–18.9]	0.579	3.99 [0.818–10.4]	0.607
IGFBP6, ng/ml	206 [180–216]	0.105	224 [196–283]	0.684	223 [200–254]	0.121
IGFBP7, ng/ml	18.2 [16.4–21.6]	0.393	19.2 [16.3–23]	0.684	20.5 [17.7–22.5]	0.673
IGF1/IGFBP1	69.7 [42.6–109]	**0.029**	111 [62.5–138]	0.393	131 [92.6–197]	0.079
IGF1/IGFBP2	3.51 [2.52–4.7]	0.218	2.5 [1.83–4.03]	**0.029**	4.01 [3.2–5.64]	0.061
IGF1/IGFBP3	2.4 [2.06–2.64]	**0.005**	2.81 [2.59–3.05]	0.631	3.02 [2.56–3.33]	**0.005**
IGF1/IGFBP5	41.2 [22.7–574]	0.075	94.1 [39.6–673]	0.353	501 [77.8–703]	0.171
IGF1/IGFBP6	2.39 [2.08–2.61]	0.247	2.38 [2.04–2.94]	0.436	2.84 [2.18–3.17]	0.475
IGF1/IGFBP7	26.2 [22.7–29.4]	0.280	28 [25.1–33.7]	1.000	29.7 [22.9–35]	0.394

*Abbreviations: CRP* C-reactive protein, *HFD* High-fat diet, *HGF* Hepatocyte growth factor, *IGF* Insulin-like growth factor, *IGFBP* Insulin-like growth factor-binding protein, *IL* Interleukin, *LFD* Low-fat diet, *MCP* Monocyte chemoattractant protein, *SHBG* Sex hormone-binding globulin, *TNF-α* Tumor necrosis factor-α, *tPAI1* Tissue plasminogen activator inhibitor 1

Values represent medians [95% CI]. Bold indicates statistical significance (*p* ≤ 0.05)

^a^Mann-Whitney *U* test between LFD and HFD-Obese

^b^Mann-Whitney *U* test between HFD-Lean and HFD-Obese

^c^Kruskal-Wallis test across three arms

### Obesity-associated transcriptomic changes

In addition to alterations in circulating biomarkers, obesity has also been associated with distinct transcriptomic changes in a variety of human tissues [87, 88]. To determine if this murine model of diet-induced obesity recapitulated these transcriptomic changes, we first developed a gene expression signature for obesity-induced changes in adipose tissues using mouse parametrial fat. To accomplish this, we collected parametrial fat from HFD-Obese and LFD (*n* = 6/arm) *MTB/TAN* mice following primary tumor regression. At a false discovery rate of 0.1, 679 genes with altered expression were identified with fold-change in expression > 1.5.

**Fig. 5:**
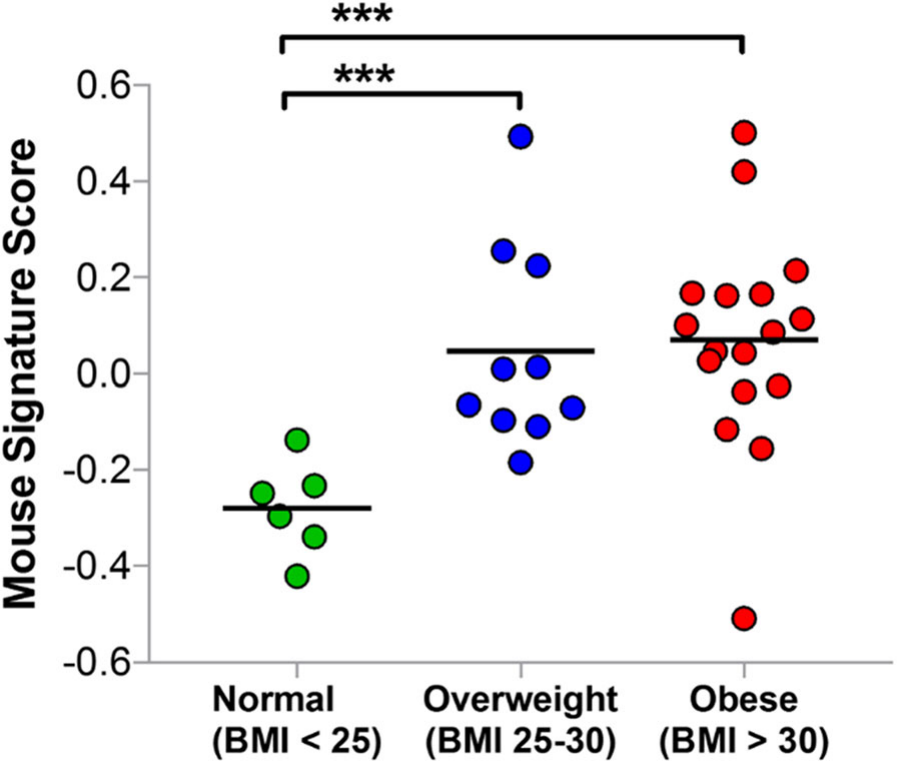
A 679-gene expression signature derived from comparison of mouse parametrial fat collected following primary tumor regression in high-fat diet (HFD)-Obese and low-fat diet (LFD) mice (fold change > 1.5, false discovery rate < 0.1) was applied to a body mass index (BMI)-stratified human dataset (Gene Expression Omnibus accession number GSE27949). Scores of an obesity-associated gene signature derived from murine adipose tissue identified parallel transcriptomic changes in human adipose samples from obese and overweight subjects compared with subjects of normal weight (overall analysis of variance *p* value = 0.003); *t* test ***p value < 0.001. Horizontal lines represent means

This 679-gene signature was applied to a BMI-stratified human gene expression dataset (GEO accession number GSE27949) derived from adipose tissue biopsies from the subcutaneous abdominal region of 33 human subjects (BMI range 24–48 kg/m^2^) [88]. Using a previously described scoring method for estimating pathway activity from gene expression data [31], this obesity-associated gene signature distinguished transcriptomic changes associated with obesity in human adipose samples (overall *p* value = 0.002) (Fig. 5). Specifically, human adipose tissue from overweight and obese individuals showed higher signature scores than adipose tissue from nonobese individuals. This suggests that adipose tissue from this mouse model exhibits obesity-associated changes in gene expression that resemble those which occur in obese human subjects.

### Obesity promotes tumor recurrence

In light of our demonstration that *MTB/TAN* mice fed a HFD develop obesity that is accompanied by multiple molecular and physiological features characteristic of human obesity, we examined the influence of diet-induced obesity on primary and recurrent mammary tumorigenesis in this model. In response to chronic HER2 expression induced by doxycycline administration, *MTB/TAN* mice developed primary breast tumors with a median primary tumor induction duration of 4.6 months. In accordance with a previous model of diet-induced obesity in which *MMTV-neu* mice were chronically exposed to a HFD [35], primary tumorigenesis was similar in obese and lean mice with no differences in mean tumor growth rate (HFD-Obese 0.277 ± 0.100 cm^3^/day vs. HFD-Lean 0.251 ± 0.104 cm^3^/day vs. LFD 0.244 ± 0.071 cm^3^/day, *p* = 0.249) or mean total tumor burden (HFD-Obese 1786.1 ± 685.9 cm^3^ vs. HFD-Lean 1929.9 ± 798.7 cm^3^ vs. LFD 1805.4 ± 639.3 cm^3^, *p* = 0.606).

To exclude the possibility of differences in doxycycline bioavailability related to changes in either dietary fat content or body weight, *MTB/TAN* mice fed either HFD or LFD were treated with doxycycline for 7 days, after which mammary tissue was collected for qRT-PCR. *HER2/neu* transgene expression levels did not vary by dietary fat composition (*p* = 0.903) (Additional file 2: Figure S2a). Because these mice were not maintained on experimental diets for a sufficient time to induce obesity, an additional subset of primary tumor-bearing HFD and LFD *MTB/TAN* mice (*n* = 5/arm) with a rate of obesity similar to that of the cohort as a whole were killed, and transgene expression was evaluated in primary tumors. Consistent with similar primary tumor burden and tumor growth rates in mice exposed to HFD or LFD, no differences in expression of *HER2/neu* transgene or total *ErbB2* were observed between experimental arms (Additional file 2: Figure S2b, c). These findings suggest that dietary fat and obesity do not influence levels of *HER2/neu* transgene expression during primary mammary tumorigenesis.

**Fig. 6:**
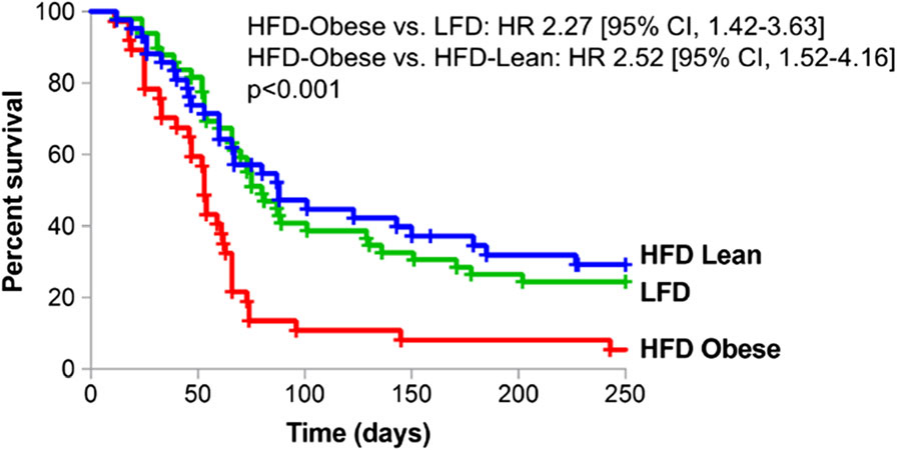
Diet-induced obesity is associated with accelerated mammary tumor recurrence (median recurrence-free survival [RFS]: high-fat diet [HFD]-Obese: 53.0 days vs. HFD-lean: 87.0 days vs. low-fat diet [LFD]: 80.0 days, *p* < 0.001) and increased recurrence risk (relative to HFD-Lean: HR 2.52, 95% CI 1.52–4.16; relative to LFD: HR 2.27, 95% CI 1.42–3.63). There was no significant difference in RFS between the two lean cohorts (*p* = 0.647)

Following oncogene downregulation induced by doxycycline withdrawal, all mice exhibited tumor regression to a nonpalpable state, after which they were monitored twice weekly for tumor recurrence. Strikingly, the rate at which mammary tumors spontaneously recurred in HFD-Obese mice was markedly accelerated compared with HFD-Lean and LFD mice (median recurrence-free survival [RFS] 53.0 days vs. 87.0 days vs. 80.0 days, log-rank *p* < 0.001) (Fig. 6). Additionally, HFD-Obese mice were more likely to develop recurrent tumors than HFD-Lean (94.6% vs. 69.0%, *p* = 0.004; HR 2.42, 95% CI 1.46–4.01) or LFD (94.6% vs. 75.5%, *p* = 0.018; HR 2.30, 95% CI 1.43–3.69) mice over 250 days of follow-up. No significant differences in RFS or recurrence incidence were observed between HFD-Lean and LFD mice. Thus, both the rate and the incidence of mammary tumor recurrence are increased in *MTB/TAN* mice as a consequence of obesity, and this effect cannot be attributed to a HFD per se.

### Obesity promotes persistence of residual tumor cells

Recurrent breast cancers in patients arise from the reservoir of residual tumor cells that survive and persist following treatment [89, 90]. In an analogous manner, the mammary glands of *MTB/TAN* mice in which tumors have regressed to a nonpalpable state following oncogene downregulation harbor a population of dormant residual tumor cells that are Ki-67-negative and bromodeoxyuridine-negative [30, 49]. We hypothesized that the increased rate and incidence of tumor recurrence observed in obese mice might be due to an obesity-induced increase in the survival and persistence of residual tumor cells following HER2 downregulation. To test this hypothesis, we injected *MTB/TAN* primary tumor cells into the mammary fat pads of immunocompetent *TAN* recipient mice maintained on a HFD or LFD. These cells were modified to constitutively expressed green fluorescent protein (GFP) to enable spatial localization of residual tumor cells within surrounding benign mammary tissue. *TAN* recipient mice were fed experimental diets for 20 weeks prior to primary tumor cell injection, which allowed for their age at oncogene de-induction to match the age of *MTB/TAN* mice at the time of primary tumor de-induction. In accordance with this, body weights of *TAN* mice were comparable to those of *MTB/TAN* mice at the time of enrollment into the recurrence assay 4 weeks following oncogene de-induction.

**Fig. 7:**
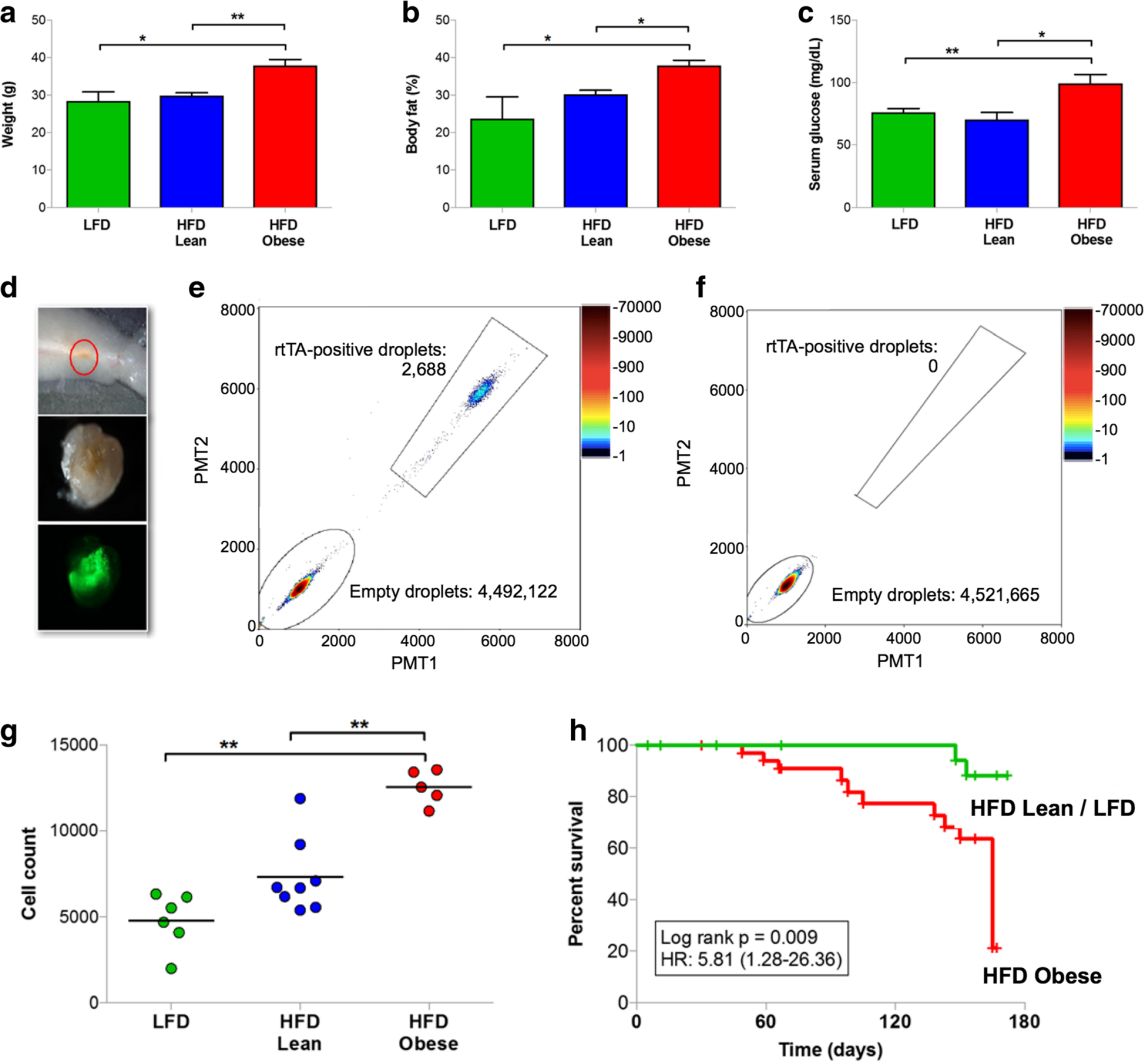
**a** High-fat diet (HFD)-Obese, HFD-Lean, and low-fat diet (LFD) *TAN* mice (*n* = 5/arm) were killed following primary tumor regression to assess residual disease. HFD-Obese mice weighed significantly more than HFD-Lean and LFD mice (37.9 ± 5.3 g vs. 29.8 ± 1.6 g vs. 28.4 ± 4.9 g, respectively; *p* = 0.004). **b** HFD-Obese *TAN* mice had elevated body fat percentage relative to HFD-Lean and LFD mice (37.9 ± 4.5% vs. 30.1 ± 2.0% vs. 23.7 ± 11.5%, respectively; *p* = 0.004). **c** HFD-Obese *TAN* mice had elevated fasting serum glucose levels relative to HFD-Lean and LFD mice (99.6 ± 15.3 mg/dl vs. 70.3 ± 10.0 mg/dl vs. 76.2 ± 6.8 mg/dl, respectively; *p* = 0.009). **d** Representative bright-field and fluorescence imaging of residual disease within the mammary glands of mice following tumor regression. **e** Representative residual lesion quantification of *rtTA*-positive droplets by droplet digital PCR (ddPCR). **f** Representative control mammary tissue quantification of *rtTA*-positive droplets by ddPCR. **g** HFD-Obese mice harbored a significantly greater number of *rtTA*-positive tumor cells in residual lesions relative to HFD-Lean and LFD mice (12,550 ± 991 vs. 7339 ± 2182 vs. 4793 ± 1618 cells, respectively; *p* < 0.001). **h** Recurrence-free survival of *TAN* mice (*n* = 15/arm) following primary tumor induction by orthotopic injection of *MTB/TAN* tumor cells and subsequent doxycycline withdrawal. Recurrence-free survival was significantly poorer for HFD-Obese mice than for HFD-Lean and LFD mice (log-rank *p* = 0.009, HR 5.81, 95% CI 1.28–26.36). Dot plots show mean data. Error bars represent the SEM. *p < 0.05, **p < 0.01

Similar to *MTB/TAN* mice, *TAN* mice exposed to a HFD became obese, as manifested by increased body weight, increased percentage of body fat, and increased fasting glucose levels compared with lean control mice (Fig. 7a–c). Following the generation of obesity with 20 weeks of exposure to a HFD, GFP-labeled *MTB/TAN* primary tumor cells were orthotopically injected into lean and obese mice, and *HER2/neu* transgene expression was induced by doxycycline administration to drive primary tumor formation, and then doxycycline was withdrawn to induce *HER2/neu* downregulation and tumor regression to a nonpalpable state. Mammary glands were then harvested, and the region of MRD was isolated using fluorescence guidance (Fig. 7d). The absolute number of residual tumor cells was then quantified by ddPCR [91] for the *rtTA* transgene performed on genomic DNA isolated from residual lesions because only *MTB/TAN* tumor cells (Fig. 7e), and not host *TAN* cells (Fig. 7f), contain this allele.

Residual lesions from HFD-Obese mice contained a significantly greater number of residual tumor cells, as reflected by the number of *rtTA* alleles, than those from HFD-Lean or LFD mice (*p* < 0.001; 12,550 ± 991 vs. 7339 ± 2182 vs. 4793 ± 1618 cells, respectively) (Fig. 7g). These data suggest that, compared with nonobese mice fed either a LFD or HFD, a greater number of residual tumor cells survive and persist in obese mice following tumor regression.

In a parallel experiment, HFD-Obese, HFD-Lean, and LFD *TAN* recipient mice harboring residual disease were followed until clinical recurrence. Consistent with our studies in intact *MTB/TAN* mice, HFD-Obese mice exhibited an accelerated rate and an increased incidence of recurrence compared with nonobese mice (log-rank *p* = 0.009; HR 5.81, 95% CI 1.28–26.36) (Fig. 7h). In aggregate, these data support a model in which the association of obesity with an increased risk of tumor recurrence is causal in nature and mediated by an obesity-induced increase in the pool of residual tumor cells that persist following tumor regression.

## Discussion

Developing more effective strategies to improve long-term survival for patients with breast cancer would be enabled by a deeper understanding of the biologic pathways governing tumor dormancy and cancer recurrence. In this regard, mechanistic studies aimed at gaining an understanding of the biological basis for the positive association between obesity and recurrence risk have been hampered by a lack of animal models. In this paper, we describe a mouse model for studying the impact of diet-induced obesity on breast cancer recurrence that recapitulates key features of the human disease. First, mice fed a HFD exhibit physiological changes analogous to those observed in humans, including increased body weight and fat mass, hyperinsulinemia, fasting hyperglycemia and impaired glucose tolerance consistent with insulin resistance, dysregulated circulating adipokine levels, and characteristic gene expression changes in adipose tissue. Second, paralleling epidemiological observations in breast cancer survivors, obese mice exhibit an increased incidence and an accelerated rate of mammary tumor recurrence. To our knowledge, this is the first experimental model for studying the impact of obesity on tumor recurrence for any cancer type. Third, our finding that diet-induced obesity accelerates breast cancer recurrence in both intact and orthotopic mouse models suggests that at least part of the effects of obesity on recurrence risk are non-cancer cell autonomous and are thus likely due to obesity-induced changes in the tumor cell microenvironment. Last, our observation that obesity results in an increased number of residual tumor cells that survive and persist following tumor regression suggests a novel hypothesis to explain the biological mechanisms by which obesity might result in accelerated tumor recurrence.

Whether the epidemiological association between obesity and decreased RFS observed in humans reflects a causal relationship, or is instead a consequence of the many nonbiological factors that can affect diagnosis and treatment in obese patients, has remained open to debate. Our observation that obese mice fed a HFD exhibit an increased rate and incidence of spontaneous tumor recurrence compared with LFD or HFD-Lean mice provides direct evidence that the relationship between obesity and risk of tumor recurrence is causal in nature. Further, our observations that obese mice experience accelerated breast cancer recurrence compared with genetically identical nonobese mice exposed to the same HFD (i.e., HFD-Lean), and that LFD and HFD-Lean mice exhibit similar rates of recurrence, strongly suggest that the increased rates and incidence of recurrence observed in HFD-Obese mice are due to obesity per se rather than to the HFD to which both HFD-Obese and HFD-Lean mice were exposed. Accordingly, this model should permit the identification of mechanisms and biomarkers for the impact of obesity on recurrence risk that are separable from those of dietary fat.

The presence of residual disease in the breast or local lymph nodes in patients with breast cancer following completion of neoadjuvant chemotherapy is highly predictive of disease relapse [55, 56]. In this regard, a striking finding of our experiments is the observation that obese mice harbor an increased number of residual tumor cells following tumor regression, compared with HFD-Lean or LFD controls, and that these increases parallel the changes in recurrence risk observed in HFD-Obese, HFD-Lean, and LFD cohorts. These data suggest that obesity may alter the dynamics of residual tumor cells by increasing the number of cells that survive and persist following treatment of the primary tumor (i.e., HER2 inhibition), such that the pool of residual tumor cells “at risk” for reentry into the cell cycle is increased and, by extension, the risk of recurrence is increased. Consistent with this hypothesis, we have previously demonstrated that genetic or pharmacological interventions that reduce the number of residual tumor cells that persist following tumor regression result in a reduced rate and incidence of tumor recurrence [30–32]. These findings provide a potential biological mechanism by which obesity may increase recurrence risk.

Ostensibly, obesity could impact recurrence risk by increasing the number of residual tumor cells that survive therapy, by enriching residual tumor cell populations for phenotypically aggressive cells that are more likely to recur, or both. Although it remains to be determined whether the increase in residual tumor cell abundance that we observed fully accounts for the obesity-induced increase in recurrence risk, or whether this increased pool of residual tumor cells is also enriched for phenotypically aggressive cells, the model we describe should enable these possibilities to be distinguished.

Studies in genetically obese and diet-induced obese rodent breast cancer models have demonstrated that obesity leads to accelerated primary tumorigenesis in models of ER+ and triple-negative (ER−PR−HER2−) breast cancer [36–38, 41, 42, 45, 47]. In contrast, HER2-overexpressing tumor models have been used only infrequently and, in agreement with human epidemiological observations, have not demonstrated an increased risk of primary tumors in the setting of obesity [35]. To our knowledge, however, rodent studies have not previously been performed to determine the impact of obesity on breast cancer recurrence.

Although obesity-induced alterations in several molecular pathways and circulating biomarkers have been hypothesized to underlie the epidemiological association between obesity and breast cancer recurrence risk, none have been proven at a functional level. In this regard, the serum biomarker findings we report provide several avenues for further experimentation. For example, obesity-induced hyperleptinemia and increases in insulin/IGF-1 levels, as observed in the present study and in other models, can lead to activation of phosphoinositide 3-kinase and mammalian target of rapamycin (mTOR) signaling and promote primary tumor growth [92]. Conversely, pharmacological blockade of the mTOR pathway has been reported to reverse these obesity-induced effects in a model of Wnt1-induced primary mammary tumorigenesis [37]. By extension, these observations suggest a potential link between obesity-induced increases in mTOR pathway activation and recurrence risk that could provide a rationale for the therapeutic use of mTOR inhibitors to prevent recurrence.

In contrast to hyperleptinemia and increased insulin/IGF-1 signaling, circulating levels of estrogen and testosterone in this mouse model did not differ significantly between obese and lean mice. Increased levels of endogenous estrogens due to aromatization of androgens in adipose tissue in obese postmenopausal women have previously been hypothesized to promote breast cancer recurrence [93, 94]. If true, obesity-induced effects on breast cancer recurrence would be predicted to be limited to ER+ breast cancer. In contrast, however, one of the largest meta-analyses of 43 primarily observational cohort studies revealed poorer breast cancer-specific survival in obese women even after adjusting for ER, PR, or HER2 overexpression [18]. In addition, it is worth noting that although the association of obesity with an increased risk of ER+ breast cancer could reflect dysregulation of the endogenous ER pathway, it could instead represent a manifestation of luminal breast cancers, which in humans are typically ER+. Insofar as the *MTB/TAN* mouse model is a model for luminal breast cancer, it is interesting to consider the possibility that the mechanisms by which obesity impacts risk of recurrence reflect the impact of obesity on the biology of luminal breast cancers.

With respect to HER2-overexpressing tumors, researchers in a phase III clinical trial (N9831) that incorporated trastuzumab adjuvant therapy for HER2+ breast cancer observed poorer breast cancer-specific survival in the setting of obesity [20]. Similar findings were observed for both ER+HER2+ tumors in a meta-analysis of eight neoadjuvant chemotherapy trials [95] and ER−HER2+ tumors in a pre-trastuzumab era study [96]. In aggregate, these findings indicate that obesity is associated with an increased risk of recurrence for HER2+ breast cancers in patients, a conclusion that is consistent with our observations in mice and that further supports the utility of this model. Additional studies are required to evaluate the applicability of these findings for specific subtypes of human breast cancer, including those that are ER+, HER2−, or triple-negative.

As noted above, rodent models to date have been used to explore the effects of obesity on breast cancer exclusively with respect to primary tumorigenesis. Accordingly, the mouse model we describe provides several advantages over previously used models. Beyond its focus on spontaneous recurrent mammary tumorigenesis, which accounts for the lion’s share of breast cancer mortality in patients, benefits of the current model include the use of immunocompetent mice, use of diet-induced rather than genetic forms of obesity, and inclusion of diet responders (i.e., HFD-Obese) and nonresponders (i.e., HFD-Lean), thereby permitting separation of effects of obesity from those of a HFD per se. Together, these characteristics provide a new tool for exploring those aspects of breast cancer progression that are responsible for the vast majority of deaths resulting from this disease.

## Conclusions

In summary, these studies provide a genetically engineered mouse model to study the association of diet induced obesity with breast cancer recurrence, which to our knowledge is the first experimental model for the impact of obesity on tumor recurrence for any cancer type. In addition to recapitulating the physiological changes characteristic of obese patients, this mouse model establishes that the association between obesity and breast cancer recurrence risk is causal in nature, and suggests that the impact of obesity on recurrence risk may be mediated by obesity-induced increases in the survival and persistence of residual tumor cells.

## Additional files

Additional file 1: **Figure S1. a** Serum glucose levels were measured after an overnight fast at time of clinical recurrence (*n* = 50/arm). Fasting glucose levels were elevated in HFD-Obese mice relative to HFD-Lean and LFD mice (129.8 ± 33.2 mg/dl vs. 118.9 ± 15.6 mg/dl vs. 114.7 ± 18.7 mg/dl, respectively; *p* < 0.001). **b** Glucose values during 2-h intraperitoneal glucose tolerance test (IP-GTT). **c** Quantification of 2-h IP-GTT using AUC_glucose_, where HFD-Obese mice had significantly higher glucose levels relative to HFD-Lean and LFD mice (23,341.7.4 ± 9202.8 mg/dl/minute vs. 17,331.2 ± 6227.8 mg/dl/minute vs. 16,747.8 ± 8681.4 mg/dl/minute, respectively; *p* < 0.001; *n* = 50/arm). Error bars represent the SEM. *p < 0.05, **p < 0.01, and ***p < 0.001. (TIFF 837 kb)

Additional file 2: **Figure S2. a** *HER2/neu* transgene expression does not vary by dietary composition following doxycycline induction for 7 days (*p* = 0.903). Transgene was not expressed in the absence of doxycycline. **b** A subset of mice (*n* = 5/arm) was killed at the time of doxycycline withdrawal, and primary tumor mRNA expression was analyzed. There were no differences in total *ErbB2* expression between study arms (analysis of variance [ANOVA] *p* value = 0.42). **c** There were no differences in transgene-specific luciferase expression between study arms (ANOVA *p* value = 0.69). Error bars represent the SEM. (TIFF 842 kb)

## Abbreviations

BMI: Body mass index
CRP: C-reactive protein
ddPCR: Droplet digital PCR
ELISA: Enzyme-linked immunosorbent assay
ER: Estrogen receptor
FAM: 6-Carboxyfluorescein
GEO: Gene Expression Omnibus
GFP: Green fluorescent protein
HER2: Human epidermal growth factor receptor 2
HFD: High-fat diet
HGF: Hepatocyte growth factor
IGF: Insulin-like growth factor
IGFBP: Insulin-like growth factor-binding protein
IL: Interleukin
IP-GTT: Intraperitoneal glucose tolerance test
LFD: Low-fat diet
MRD: Minimal residual disease
mTOR: Mammalian target of rapamycin
PR: Progesterone receptor
RFS: Recurrence-free survival
RNA-Seq: RNA sequencing
SHBG: Sex hormone-binding globulin
Simoa: Single-molecule array
TNF-α: Tumor necrosis factor-α
tPAI1: Tissue plasminogen activator inhibitor 1
