# Supplementary figures and images for: Impact of obesity on breast cancer recurrence and minimal residual disease
Source: Breast Cancer Res. 2019 Mar 13;21:41. doi: 10.1186/s13058-018-1087-7 (PMC6416940; doi:10.1186/s13058-018-1087-7)

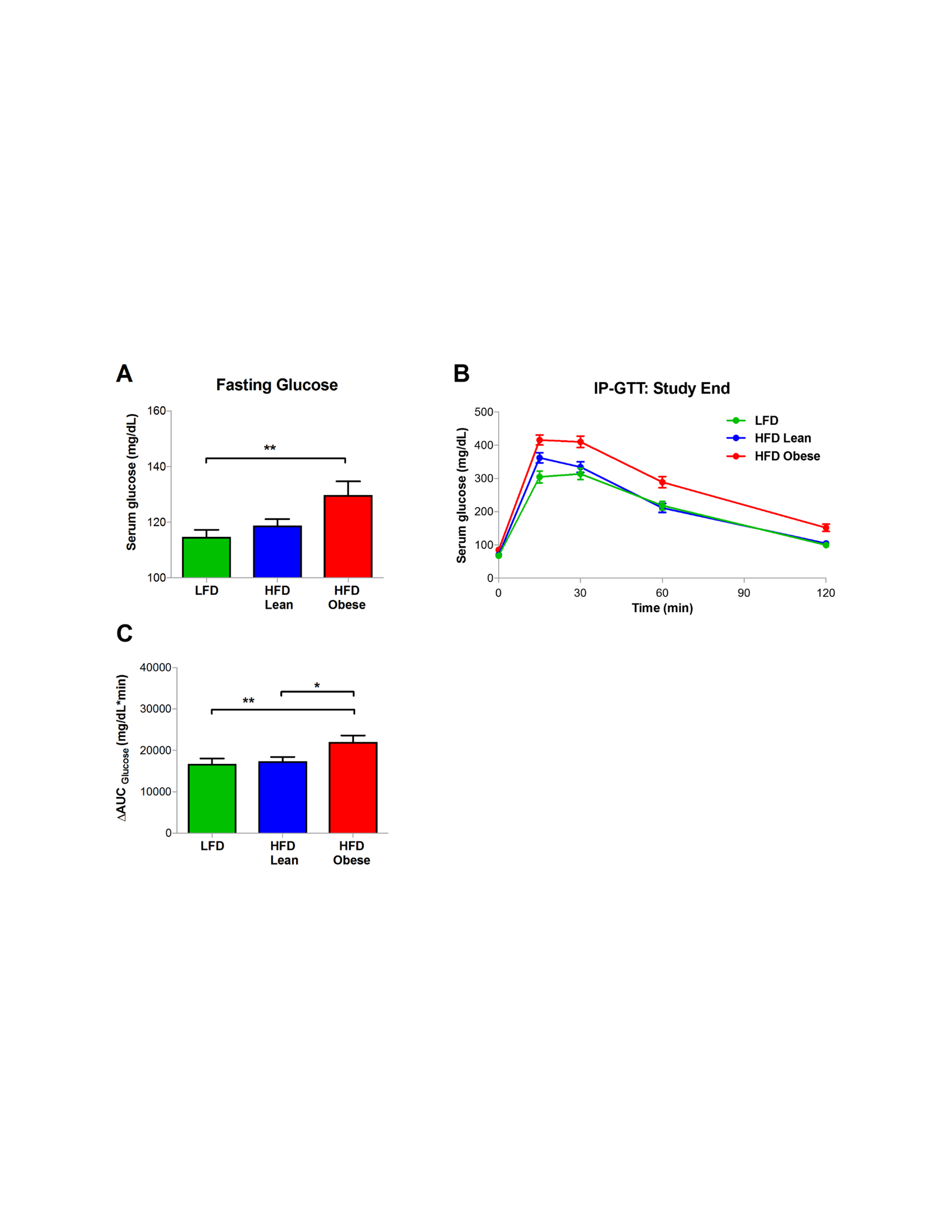

Supplement: Supplementary file 1 — Figure S1. a Serum glucose levels were measured after an overnight fast at time of clinical recurrence (n = 50/arm). Fasting glucose levels were elevated in HFD-Obese mice relative to HFD-Lean and LFD mice (129.8 ± 33.2 mg/dl vs. 118.9 ± 15.6 mg/dl vs. 114.7 ± 18.7 mg/dl, respectively; p < 0.001). b Glucose values during 2-h intraperitoneal glucose tolerance test (IP-GTT). c Quantification of 2-h IP-GTT using AUCglucose, where HFD-Obese mice had significantly higher glucose levels relative to HFD-Lean and LFD mice (23,341.7.4 ± 9202.8 mg/dl/minute vs. 17,331.2 ± 6227.8 mg/dl/minute vs. 16,747.8 ± 8681.4 mg/dl/minute, respectively; p < 0.001; n = 50/arm). Error bars represent the SEM. *p < 0.05, **p < 0.01, and ***p < 0.001. (TIFF 837 kb) [file 13058_2018_1087_MOESM1_ESM.tif]

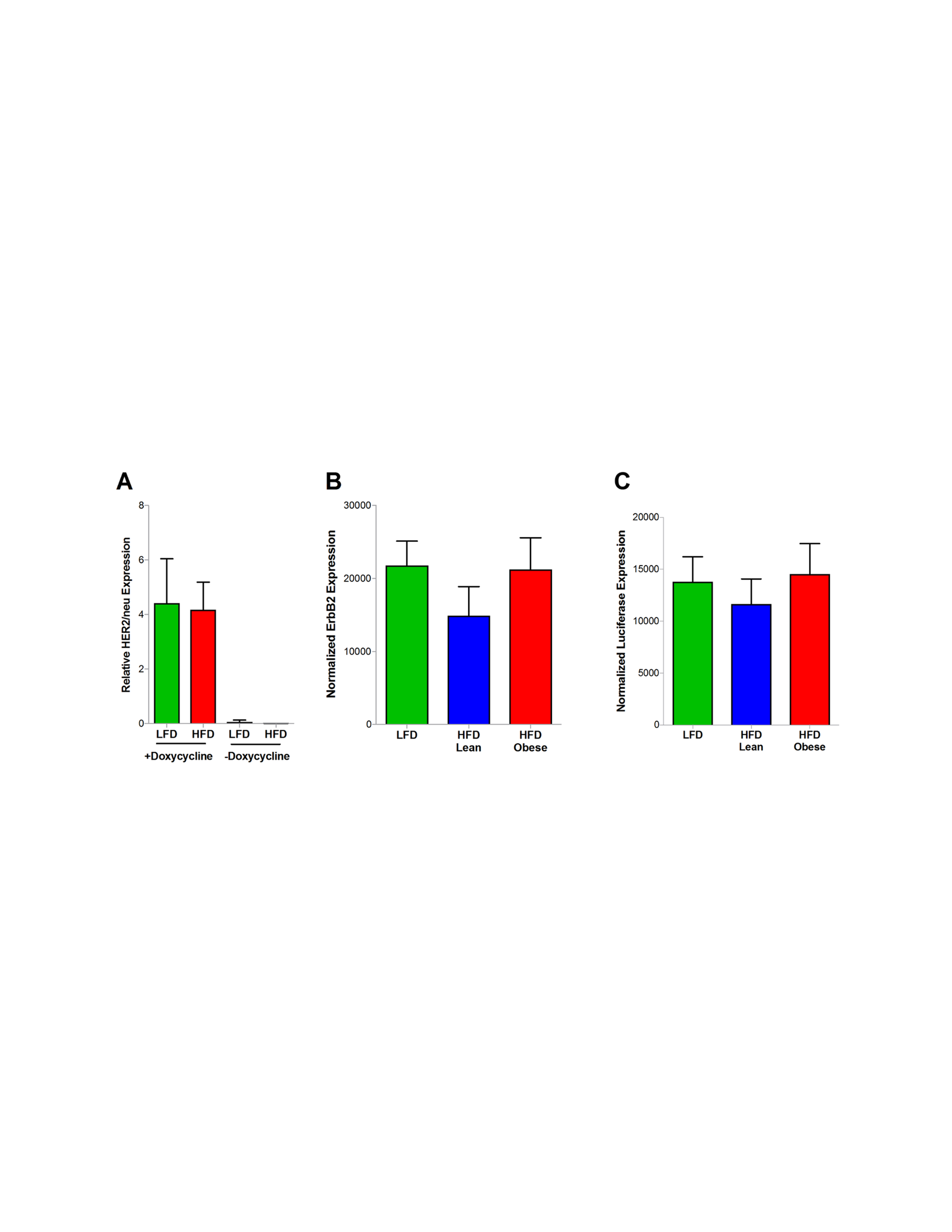

Supplement: Supplementary file 2 — Figure S2. a HER2/neu transgene expression does not vary by dietary composition following doxycycline induction for 7 days (p = 0.903). Transgene was not expressed in the absence of doxycycline. b A subset of mice (n = 5/arm) was killed at the time of doxycycline withdrawal, and primary tumor mRNA expression was analyzed. There were no differences in total ErbB2 expression between study arms (analysis of variance [ANOVA] p value = 0.42). c There were no differences in transgene-specific luciferase expression between study arms (ANOVA p value = 0.69). Error bars represent the SEM. (TIFF 842 kb) [file 13058_2018_1087_MOESM2_ESM.tif]
